# Gelatin Adsorption onto Cellulose Nanocrystals Surfaces at Different pH: A QCM‑D Study

**DOI:** 10.1021/acs.langmuir.5c00795

**Published:** 2025-06-10

**Authors:** Jessica Borges-Vilches, Tuuli Virkkala, Kristoffer Meinander, Ilkka Kilpeläinen, Tekla Tammelin, Eero Kontturi

**Affiliations:** † Department of Bioproducts and Biosystems, 174277 Aalto University, FI-00076 Aalto, Finland; ‡ 3259 VTT Technical Research Centre of Finland Ltd, VTT, P.O. Box 1000, FI-02044 Espoo, Finland; § Department of Chemistry, Material Division, University of Helsinki, FI-00560 Helsinki, Finland

## Abstract

By exploiting the pH-responsive behavior of gelatin, this study investigates the influence of pH on gelatin’s properties both in solution and when adsorbed on cellulose nanocrystal (CNC) surfaces. To ensure a broad exploration of this system, the study was carried out below (pH 5), above (pH 11), and at the isoelectric point of gelatin (pH 8). In solution, gelatin exhibited strong pH-dependent behavior, with hydrodynamic diameters increasing from 15.7 nm at pH 5 to 27.9 nm at pH 8, and ζ-potential varying from 12.4 mV to -10.9 mV as pH shifted from 5 to 11. However, Nuclear Magnetic Resonance analysis revealed that gelatin does not undergo conformational changes in its secondary structure, suggesting that gelatin’s pH responsiveness in solution is driven by self-aggregation or interactions with other polymers rather than conformational changes of the gelatin molecule itself. When adsorbed onto CNCs, gelatin showed a markedly different behavior. At pH 8, the frequency change observed in Quartz Crystal Microbalance with Dissipation (QCM-D) was 5–6 times higher than at pH 5 or 11, indicating greater adsorption, whereas dissipation changes were also 2–3 times higher at pH 8 than its counterparts. The reduction in surface charge and solubility of gelatin at its isoelectric point minimizes water release during adsorption, allowing more gelatin to bind to CNCs. At pH 5 and 11, when gelatin behaves as a polyelectrolyte, similar frequency and dissipation shifts suggest an adsorption mechanism primarily driven by entropic gain. pH also strongly affected the viscoelastic interfacial properties of CNC surfaces with adsorbed gelatin, with hydrodynamic thicknesses at pH 5 and 11 being smaller than the gelatin diameter in solution, indicating molecular reorientation of surface-bound gelatin molecules. Despite differing behaviors in solution and on the CNC surface, both scenarios suggest the presence of extended gelatin chains rather than globular structures under all pH conditions. These findings enhance understanding of pH-dependent gelatin behavior and offer insights for designing responsive nanostructured materials.

## Graphical abstract

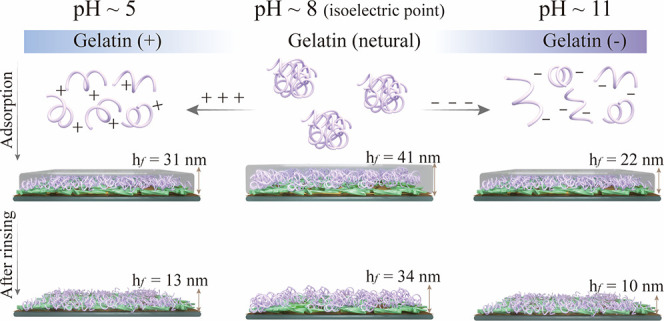

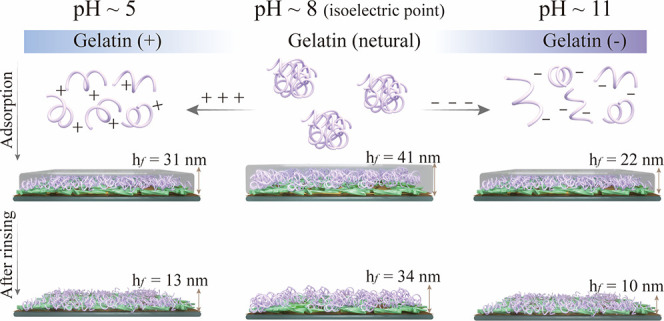

## 1 Introduction

Understanding how complex biomolecules interact with nanomaterial surfaces underlies the functionality and performance of nanomaterials in a wide range of applications, including protein adsorption, food processing, biosensors, and biomedicine.^1,2^ In particular, the protein-nanomaterial interactions are of great importance in biomedicine,^3–6^ where controlled protein deposition and release on medical implants or during healing processes can enhance the compatibility with biological surfaces, accelerate recovery, and minimize side effects.^7,8^ In this study, we explore the adsorption of one of the most common proteins (gelatin) on ultrathin films of biologically derived nanoparticles (cellulose nanocrystals (CNCs)).

Among the natural polymers used in biomedical applications, gelatin is one of the most widely utilized due to its ability to mimic the essential functions and properties of an extracellular matrix. Gelatin is a naturally derived polymer obtained from the partial hydrolysis and denaturation of collagen, the most abundant component of the extracellular matrix. It is biocompatible, biodegradable, and non-toxic.^9,10^ Furthermore, gelatin is responsive to pH and temperature, which makes it particularly versatile in the biomedical realm. Despite its advantageous properties, gelatin has inherent drawbacks, such as low mechanical stability, high degradation rates, and limited durability, which restricts its wider application.^11,12^ Combining gelatin with biocompatible polymers has proven to be an effective strategy to overcome these limitations, thereby improving its reactivity and overall functionality of gelatin-based composites.^9,13^

CNCs, in turn, are rigid nanorods derived from plant fibers.^14^ They have attracted considerable attention in polymer science as nanofillers due to their high mechanical strength, large specific surface area, biodegradability, biocompatibility, and non-toxicity.^15,16^ Independent of the application area, which ranges from packaging composites to biomedical hydrogels,^4,5,16,17^ CNCs are seldom applied on their own: they often form one component in a composite material. For example, cross-linking CNCs with gelatin to form hydrogels has been shown to improve the overall performance of composites by combining the beneficial properties of both materials.^4,5,10^

Although previous studies have explored the surface interactions between CNC and gelatin, they have largely overlooked a comprehensive analysis of gelatin’s solution properties and their role in governing adsorption onto CNC surfaces. In particular, the interplay between the solution environment, gelatin adsorption behavior, and the interfacial rheological properties of the adsorbed gelatin layer has not been systematically investigated, despite the efforts in surface-sensitive studies on systems containing cellulose and proteins.^17,18^ The novelty of our approach lies in clearly distinguishing between the intrinsic solution properties of gelatin and the characteristics of its adsorbed layers on CNCs. By applying the Voigt model, we provide a quantitative understanding of the interfacial rheological features of these adsorbed layers—an aspect that has been largely unexplored in such systems. Furthermore, our findings challenge the conventional paradigm that protein adsorption is predominantly driven by electrostatic interactions, hydrogen bonding, or hydrophobic effects. Instead, we show that adsorption of gelatin under different pH conditions is not governed by the much-touted conformational change, and the electrostatics play a smaller role than in most cases where cellulose surfaces and polyelectrolytes are involved.

In this study, we present a straightforward approach to modulating gelatin adsorption on cellulosic materials using an experimental model system composed of ultrathin films of carboxylated cellulose nanocrystals (CNCs), in which the adsorption of type A gelatin was monitored in situ via Quartz Crystal Microbalance with Dissipation monitoring (QCM-D) under three distinct pH conditions: below (pH 5), at the isoelectric point (pH 8), and above (pH 11). The condition of pH 5 was selected to investigate protonation effects in the gelatin molecule under mildly acidic conditions, without risking protein degradation or denaturation. Furthermore, pH 8 and pH 11 were chosen to further study gelatin behavior when it carries no surface charge (pH 8) and when it becomes a negatively charged polymer (pH 11). These findings provide a deeper mechanistic understanding of protein–polysaccharide interactions and offer a valuable framework for designing CNC-based biomaterials with tailored surface properties for applications such as biomedical devices or smart responsive surface systems.

## 2 Materials and Methods

### 2.1 Materials

Gelatin (type A gelatin derived from acid-cured porcine skin, gel strength Bloom No. 300) was purchased from Sigma-Aldrich. The product information sheet indicates that type A gelatin has 78–80 mM free carboxyl groups per 100 g of protein. Other chemicals such as sodium hydroxide solution (NaOH, 0.1N), hydrogen chloride (HCl gas, 99.8%), sodium hypochlorite solution (NaOCl, 6–14% active chlorine), sodium chloride (NaCl, ≥99.0%), and 2,2,6,6-tetramethylpiperidine 1-oxyl, 2,2,6,6-tetramethyl-1-piperidinyloxy (TEMPO, free radical) were also purchased from Sigma-Aldrich. A bacterial cellulose flake substrate (Sigma-Aldrich) was used as raw material for the CNC preparation. Poly­(ethylene imine) solution in water (PEI, 33 wt %, *M*_w_ = 50,000–100,000) was purchased from Polysciences Inc. (Warrington, PA, USA). Deuterium oxide (D_2_O, 99.9%) was purchased from Euroisotop. AT-cut gold-coated quartz crystal sensors (QSX 301) were obtained from Biolin Scientific AB, Sweden. The sensors have a fundamental resonance frequency *f*_0_ = 5 MHz and a sensitivity constant *c* = 0.177 mg m^–2^ Hz^–1^, as reported by the supplier. Milli-Q water (18.2 MΩ cm resistivity) and deionized water (DW) were used throughout the investigation. All reagents and solvents were of analytical grade and used as received.

### 2.2 CNC Production

Carboxylated CNCs were prepared from a bacterial cellulose substrate using a well-established and thoroughly characterized protocol developed in our group, based on the procedure proposed by Pääkkönen et al.^19^ A reference AFM image of the CNCs, including their length and height distributions, is shown in Figure S1, Supporting Information. The resulting CNCs had a length of 125 ± 32 nm, height of 11.2 ± 2.8 nm, and aspect ratio of 11.2 nm, as measured using AFM images.

The carboxylate content of the resulting CNCs was measured using conductometric titration in accordance with SCAN-CM 65:02. A wet, washed sample obtained after TEMPO-oxidation —equivalent to 300 mg of dry cellulose—was dispersed in 500 mL of degassed Milli-Q water and 0.5 mL of 0.5 M NaCl solution. The mixture was acidified by adding 5 mL of 0.1 M HCl, followed by titration with 20 mL of 0.1 M NaOH at a rate of 0.1 mL min^-1^. Three independent measurements were performed, and an average surface charge of 0.96 ± 0.04 mmol COOH/g sample was obtained.

The amount of negatively charged carboxylate groups present on the CNC surface at pH 5 was also determined by pH and conductometric titrations. The protocol and results of these titrations are provided in the Supporting Information, and the results are shown in Figure S2.

### 2.3 Dynamic Light Scattering and ζ-Potential Measurements

The hydrodynamic size and surface charge of gelatin solutions prepared at pH 5, 8, and 11 were evaluated by Dynamic Light Scattering (DLS) and ζ-potential measurements, respectively. A gelatin solution stock with a 0.05 wt % concentration was prepared in Milli-Q water under constant stirring at 45 °C. The pH of the gelatin solutions was adjusted to 8 and 11 with 0.5 M NaOH solution, while pH 5 was obtained directly after gelatin dissolution. The solutions were filtered through a 0.45 μm membrane filter to remove large, undissolved particles. All measurements were performed at room temperature in a Malvern Zetasizer Nano ZS90 device. A scatter angle of 173° was used for ζ-potential measurements. Four replicas were performed for each sample to ensure reproducibility and reliability of the measurements.

The ζ-potential of the CNCs under the pH conditions studied was also determined. A 0.05 wt % CNCs suspension was prepared using Milli-Q water. The pH of the CNCs suspensions was adjusted to 5 using 0.1 M HCl, while the pH 8 and 11 were adjusted by adding 0.1 M NaOH. The CNC suspensions containing 250 μL of 10 mM NaCl were prepared at each pH condition in a similar way as previously described. After adjusting pH, each suspension was stirred for 20 min, filtered with a 0.45 μm membrane filter and measured at room temperature in the zeta potential analyzer (Anton Paar Litesizer 500 device). The addition of salts was necessary to accurately measure zeta potential, as it limits the extent of the electrical double layer around the CNCs.^20^ The results are presented in Table S1 and discussed in the Supporting Information.

### 2.4 Proton Nuclear Magnetic Resonance (NMR) Analysis

The possible conformational changes in the gelatin chains as a function of pH were assessed with NMR. A gelatin solution stock with a 27 mg/mL concentration was prepared using 90:10 H_2_O/D_2_O solution at 45 °C. For the final set of NMR data, three aliquots of the stock solution were diluted 1:5 with 90:10 H_2_O/D_2_O and their pH was adjusted to 5, 8, and 11 with 0.5 M NaOH (pH 5 sample was obtained directly after gelatin dissolution). 0.5 mL of each solution was added to the NMR tubes and kept at 45 °C until analysis to avoid sample solidification. To test possible aggregation and evaluate spectral quality at different gelatin concentrations, ^1^H NMR spectra of the stock solution samples were first compared to 1:5 diluted samples at the same pH conditions. The ^1^H NMR spectra between both concentrations were identical at different pH conditions. As the 1:5 diluted sample (i.e., final gelatin concentration 5.4 mg/mL in 90:10 H_2_O/D_2_O) provided adequate signal-to-noise information, they were selected for recording the final set of the NMR data.

The NMR spectra were measured at 45 °C on Bruker Avance Neo 600 spectrometer (^1^H-frequency 600 MHz) equipped with a 5 mm triple resonance (^1^H/19F, ^13^C, ^31^P) inverse-detection probe with triple axis gradients. All NMR spectra were acquired and processed using Topspin 4.0.4-software. The ^1^H NMR spectra were recorded with 1D NOESY pulse program (noesygppr1d for efficient suppression of water signal) with a spectral width of 9615 Hz (16 ppm, transmitter at water frequency of 4.7 ppm), collection time 8 s, 8 scans (4 dummy scans), and with repetition time of 10 s. The ^1^H–^13^C HSQC spectra were recorded with standard Bruker echo/antiecho pulse program (hsqcetgp) with ^1^H spectral width of 7800 Hz (13 ppm, transmitter at 0.70 ppm) and 24,882 Hz (165 ppm, transmitter at 75 ppm) for ^13^C. The number of scans was 128, with a 1.5 s relaxation time between scans. For the indirect ^13^C dimension, 150-time increments (total number of indirectly detected points 300) were collected.

### 2.5 Study of the Gelatin-CNC Interfacial Interactions

#### 2.5.1 Preparation of CNC Films for Adsorption Studies

CNC films were prepared following a slightly modified procedure described by Eronen et al.^21^ and Hakalahti et al.^22^ Briefly, the gold sensors were rinsed with Milli-Q water, dried with nitrogen gas, and cleaned using UV/ozone (UV/ozone ProCleaner, BioForce Nanosciences Inc., Ames, IA, USA) for about 15 min. Before the deposition of CNCs, an anchoring polymer (0.1 wt % PEI) was deposited on the sensor surfaces by adsorbing for 30 min, after which the sensors were rinsed with Milli-Q and dried with nitrogen gas. The CNC suspension was diluted to 0.1 wt % using Milli-Q water and ultrasonicated with a Branson Digital Sonifier at 25% amplitude for 2 min (energy: 2 J/g for 2 mL of 0.1 wt % CNC suspension). 200 μL of CNC suspension was dispensed on the PEI-coated sensors and spin-coated at 3000 rpm for 1.5 min using a WS-400BZ-6NPP/Lite spin-coater (Laurell Technologies Corporation, North Wales, PA, USA). Lastly, the CNC-coated sensors were annealed for 10 min at 80 °C to ensure CNC attachment and stability of the surfaces during adsorption experiments.

#### 2.5.2 Adsorption of Gelatin on CNCs at Different pH via QCM-D

QCM-D (E4, Q-Sense AB, Sweden) was used to study the effect of pH on the interfacial interactions between gelatin and CNCs. In QCM-D, a piezoelectric quartz crystal sensor is placed in a pulsating electric field, causing the sensor crystal to oscillate at a specific fundamental resonance frequency (*f*_0_) and its overtones. An increase in the total mass on the sensor during adsorption causes the resonance frequency to change to *f*. Given that the adsorbed layer is rigidly adhered, can be considered even in distribution, fully elastic, and minor in mass compared to the sensor crystal, the frequency change Δ*f* (*f* – *f*_0_) is directly proportional to a change in areal mass according to the Sauerbrey eq (eq 1)­

1 $$\Delta m=-C\times \frac{\Delta f}{n}$$

where Δ*m* is the mass change per unit area, *C* is the sensitivity constant of the sensor, and *n* is the overtone number (*n* = 1, 3, 5, 7, 9, 11, 13).

During frequency monitoring, the voltage is periodically cut off, resulting in frictional losses in the adsorbed adlayer that cause the oscillation amplitude to dampen with a decay rate dependent on the viscoelastic properties of the material. The energy dissipation factor is given by eq 2 as follows

2 $$D=\frac{E_{dissipation}}{2\pi E_{storage}}$$

where *E*_dissipation_ and *E*_storage_ represent the energy lost into heat and the total energy stored during a single cycle of oscillation, respectively. Measuring several frequencies and the change in dissipation factor enables qualitative estimation of whether the adsorbed layer is rigid or soft (water-rich). The change in dissipation factor is defined as Δ*D* = *D* – *D*_0_, where *D*_0_ is the dissipation factor of the pure sensor crystal immersed in the solvent. For fully elastic layers, Δ*D* < 1 × 10^–6^, and the overtones of Δ*f* and Δ*D* do not spread significantly.

In the QCM-D measurements, the CNC surfaces were stabilized overnight in water with pH adjusted to 5, 8, and 11. A concentration of 0.5 wt % was chosen to prepare the gelatin solutions to ensure we are safely in the plateau regime of the adsorption isotherm during the experiments. The gelatin solutions were prepared by dissolving gelatin powder in Milli-Q water at 45 °C. 0.5 M NaOH solution was used to adjust the pH of the gelatin solutions to values of 8 and 11. A gelatin solution with pH 5 was obtained directly after dissolution. The final concentration of the gelatin solutions was unaffected by pH adjustment, as a relatively strong base was used to achieve the desired pH. Each solution was filtered through a 0.45 μm membrane filter to remove any aggregates before measurements. During the QCM-D measurements, a steady baseline for the CNC surfaces in water was first obtained for about 15 min at 23 °C and 0.1 mL min^–1^ flow rate. Then, the appropriate gelatin solution was injected into the flow cells, and changes in frequency (Δ*f*) and dissipation (Δ*D*) were monitored for about 60 min until equilibrium or very slow adsorption was observed. After gelatin adsorption, the surfaces were rinsed with pH-adjusted water and then rinsed with fresh Milli-Q water (no pH adjustment). The data for each overtone was measured, and only the third overtone (15 MHz, *f*_0_ = 5 MHz, *n* = 3) was used for plotting. At least three sensors were measured for each pH condition to ensure reproducibility and reliability of the measurements.

#### 2.5.3 Amount of Adsorbed Gelatin on CNC Surfaces

The amount of adsorbed gelatin on CNC surfaces was determined according to the model proposed by Johannsmann et al.^23^ According to eq 3, the shift in complex frequency (*δ̂f*) is related to the crystal’s resonance frequency in solution in the following manner

$$\mathrm{\delta ̂}f\approx -f_{0}\frac{1}{\pi \sqrt{\rho_{q}\mu_{q}}}\left(f\rho d+ĵ\left(f\right)\frac{f^{3}\rho^{3}d^{3}}{3}\right)$$ 3

where *f* is the resonance frequency of the crystal in contact with the solution, *d* is the thickness of the film, and *ĵ*(*f*) is the complex shear compliance. The values of *f*_0_, ρ_*q*_, and μ_*q*_ are constants for quartz: *f*_0_ = 5 MHz is the fundamental resonance frequency of the quartz crystal in air, ρ_*q*_ = 2648 kg m^–3^ is the specific density, and μ_*q*_ = 2.95 × 10^10^ kg m^–1^ s^–2^ is the elastic shear modulus of the quartz crystal. In terms of equivalent mass (m*), this model can be expressed as eq 4.

4 $$m^{*}=-\frac{\sqrt{\rho_{q}\mu_{q}}}{2f_{0}}\frac{\delta^{f}}{f}$$ 

Equation 4 can be rewritten in a linear form

5 $$m^{*}=m^{0}\left(1+ĵ\left(f\right)\frac{f^{2}d^{2}\rho }{3}\right)$$

In eq 5, *ĵ*(*f*) is assumed to be independent of frequency in the accessible range, and the true sensed mass (*m* ^0^) is obtained graphically as the “*y*” intercept of a plot of the equivalent mass against the square of the resonance frequency *f*_2_. Here, the third, fifth, and seventh overtones were used for the modeling, and an example of the calculations is available in the Supporting Information. It should be noted that the true sensed masses calculated by the Johannsmann model include both the dry adsorbed gelatin and the water mass associated with the adsorbed layer; therefore, these values do not represent the dry mass of adsorbed gelatin on CNC.

To investigate the possible contribution of bulk effects on QCM-D response, the kinematic and dynamic viscosities of gelatin solutions at different pH were determined. The detailed protocol of these measurements is described in the Supporting Information.

### 2.6 Surface Characterization of Gelatin-CNC Thin Films after Adsorption

The topography of the neat CNC film and gelatin-CNC thin films after adsorption was analyzed by AFM (MultiMode 8 AFM, Bruker). Surface areas of 5 × 5 μm^2^ were scanned in air-tapping mode with a resonance frequency of around 294 kHz at room temperature. AFM images were analyzed with NanoScope Analysis 3.0 (Bruker) software, and no other treatment except flattening was used on the images. The surface’s root-mean-square roughness (*R*_*q*_) values were also obtained from the same software.

The surface composition of the neat CNC film and gelatin-CNC thin films after adsorption was investigated using X-ray Photoelectron Spectroscopy (XPS). The measurements were performed in an XPS (Kratos AXIS Ultra, DLD detector) instrument using a monochromated Al Kα X-ray source (1486.7 eV) and operating at 100 W. A pass energy of 80 eV and a step size of 1.0 eV were used for the survey spectra, while a pass energy of 20 eV and a step size of 0.1 eV were used for the high-resolution spectra. Photoelectrons were collected at a 90° take-off angle under ultrahigh vacuum conditions, with a base pressure typically below 1 × 10^–9^ Torr. The diameter of the beam spot from the X-ray was 1 mm, while the area of analysis was 300 × 700 μm^2^. Survey spectra, as well as high-resolution spectra for carbon (C 1s), oxygen (O 1s), and nitrogen (N 1s), were collected from three different spots on each sample surface and shown in Figure S3, Supporting Information.

### 2.7 Interfacial Rheology of Gelatin-CNC Thin Films–Voigt Model

The interfacial rheological properties of the self-assembled gelatin-CNC thin films after adsorption were investigated using the Voigt model. As described by Voinova et al.,^24^ the Voigt model is commonly used to analyze the viscoelastic properties of soft, hydrated films adsorbed onto a sensor surface. This model characterizes the viscoelastic properties of an adsorbed layer on rigid surfaces within purely viscous and Newtonian solvents. It assumes that the thickness and layer density are uniform, the viscoelastic properties are frequency-independent, and that no slip occurs between the adsorbed layer and the crystal during shearing. The complex shear modulus of the formed layer is defined by eq 6 as follows

6 $$G=G^{\prime }+iG^{\prime }=\mu_{f}+2\pi if\eta_{f}=\mu_{f}\left(1+2\pi if\tau_{f}\right)$$

where *f* is the oscillation frequency, and τ_*f*_ is the characteristic relaxation time of the film. In this study, the evolution of the shear viscosities (η_*f*_), shear elastic moduli (μ_*f*_), and hydrodynamic thickness (*h*_*f*_) of thin films was investigated until the end of step II. In the fitting, the changes in frequency and dissipation factors were fitted to the model at 15, 25, 35, and 45 MHz (i.e., third, fifth, seventh, and ninth overtones) using the *D*_find_ program provided by Biolin Scientific AB. The density of the adsorbed gelatin layers was assumed to be 1.3 g cm^–3^ based on previous determinations of the specific volume of proteins in aqueous buffer (0.77 mL/g).^25,26^ The fitting of the Voigt model to gelatin-CNC adsorption data is shown in Figure S4, Supporting Information.

### 2.8 Statistical Analysis

Data were analyzed using OriginPro 2024 software. Statistical analysis was conducted using analysis of variance (ANOVA) and Tukey’s multiple comparisons with Statgraphics 19 Centurion software. A *p*-value ≤ 0.05 was considered statistically significant. Results were obtained from three independent experiments unless otherwise stated in the text. Results are presented as mean values ±standard deviation (SD), with error bars displayed in each figure.

## 3 Results and Discussion

### 3.1 Solution Properties of Gelatin

The effect of pH on gelatin solutions’ surface charge and hydrodynamic size was evaluated by ζ-potential and DLS measurements, respectively. As expected, the ζ-potential of gelatin significantly varied with pH (Table 1), shifting from positive to negative values with increasing pH, demonstrating the amphoteric nature of gelatin. A surface charge of −0.04 ± 0.4 mV was observed in the gelatin solution prepared at pH 8, suggesting that gelatin’s ieP is around 8, which agrees with earlier studies.^9,27^

**Table 1:** ζ-potential and Hydrodynamic Diameter of Gelatin Solutions Under Different pH Conditions

pH of the gelatin solutions	ζ-potential (mV)	mean hydrodynamic diameter (nm)
5	12.4 ± 0.7	15.7
8	–0.04 ± 0.4	27.9
11	–10.9 ± 0.2	19.1

The hydrodynamic diameter of the gelatin solutions at different pH was determined using DLS, as shown in Figure 1A. Our DLS results, presented as a function of the volume particle size distribution (PSD), were determined based on the Mie theory.^28^ This theory provides a more accurate representation of PSD by accounting for the actual number or volume of particles rather than just their scattering intensity. It considers the optical properties of the materials, such as the refractive index and the absorption of light by the material, together with the scattered intensity in its autocorrelation function, thus being the standard procedure for reporting hydrodynamic size in such studies.

**Figure 1:**
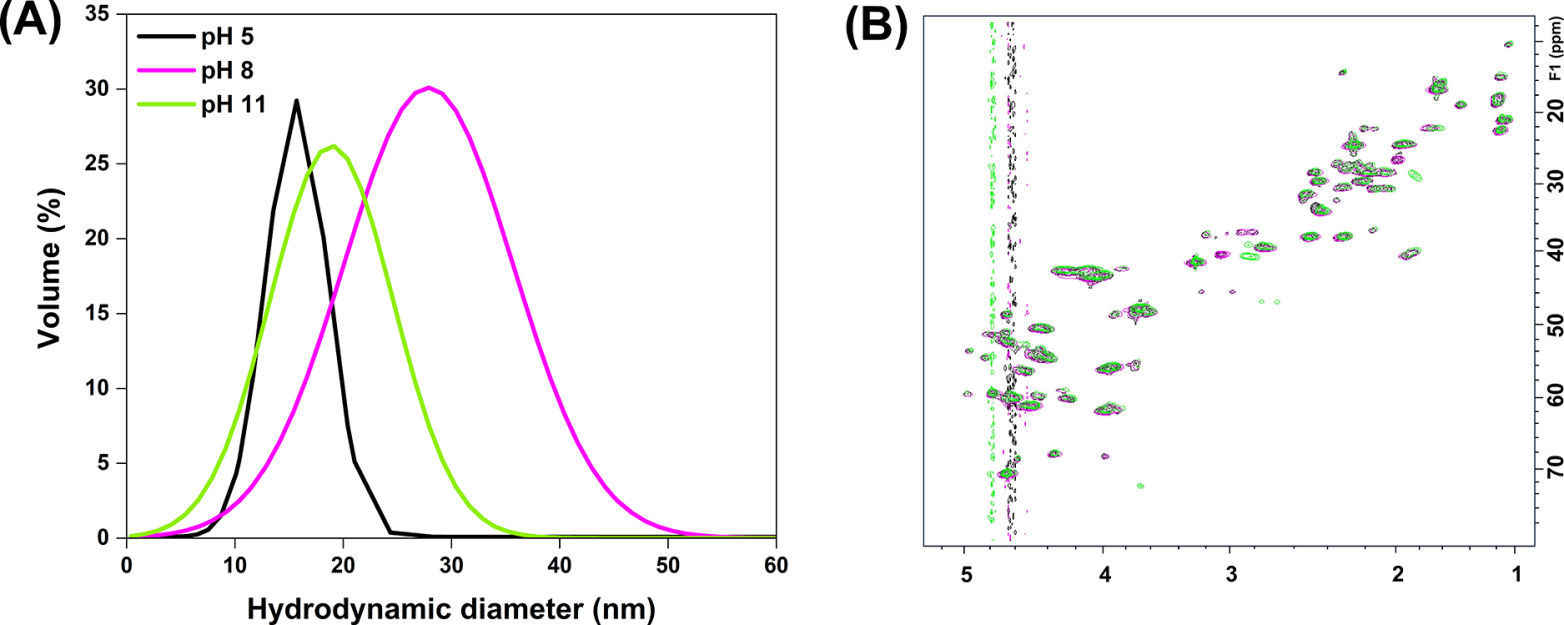
(A) Hydrodynamic diameter of gelatin solutions at pH 5, 8, and 11, (B) ^1^H–^13^C 2D HSQC spectra of gelatin. An expansion of the aliphatic chain area of the 2D HSQC spectra of gelatin overlaid at different pH conditions: pH 5 (black), pH 8 (pink), and pH 11 (green).

The results show that the gelatin’s hydrodynamic diameter significantly varied with pH, with the largest diameter observed at pH ∼ ieP (Table 1), followed by the gelatin at pH 11 and 5, respectively. This abrupt increase in size close to the gelatin’s ieP could be related to the significant reduction in gelatin’s electrostatic charges at this pH, which may facilitate protein aggregation, leading to the formation of larger gelatin clusters and, consequently, an increase in size. The fact that the hydration state of proteins is reduced as pH approaches ieP, as previously reported in the literature,^25^ also supports that the larger diameter of gelatin at pH 8 is likely due to the self-aggregation of this protein.

The possible conformational changes in the gelatin structure as a function of pH were investigated using NMR. This technique is sensitive to detecting changes in the protein’s secondary structure. As NMR chemical shifts are sensitive to the changes in the secondary structure of proteins, even slight differences in dynamic populations will introduce visible changes in the ^1^H–^13^C correlation (HSQC) spectra, especially for Cα and Cβ resonances. Inspired by this, we choose to record ^1^H–^13^C correlation spectra for gelatin under different pHs (Figure 1B). Somewhat surprisingly, there were no changes in the gelatin ^1^H–^13^C HSQC NMR spectra at different pHs, except for the basic residues, which agrees with an earlier study.^9^ The recorded spectra were of good quality in all cases (no disappearance or detectable broadening of signals), and the spectra remained identical, i.e., nicely overlapping with each other in all cases. The obtained NMR data thus strongly suggest that when gelatin is in solution, it does not undergo the so-called “coil–globule transition” at the pH conditions studied, and changes only take place at the protonation degree of the gelatin (acidic) side chains.

In proteins, the transition from random coil to α-helical conformation introduces a positive secondary chemical shift for Cα, while the transition to β-sheet conformation introduces negative secondary shifts for Cα. The transition from a random coil toward an ordered secondary structure (or vice versa) has a strong correlation to the chemical shifts of the Cα and Cβ atoms.^29,30^ Given the pH-responsive behavior of proteins, previous studies have suggested that gelatin undergoes structural/conformational transitions from random coil to α-helix due to changes in its balance of charges at different pH conditions.^9,31–34^ It has been stated that as the pH deviates from gelatin’s ieP, an increase in either positive or negative charge disrupts the stability of the helical structure of gelatin. This destabilization has been postulated to originate from the increased repulsion among similarly charged regions, preventing the gelatin molecule from folding into the stable helix structure and promoting a random coil configuration. Conversely, when the pH is near gelatin’s ieP, the balanced surface charge distribution allows gelatin molecules to interact more closely, favoring the formation of an ordered helix structure. However, this reasoning is usually based on the physical behavior of gelatin solutions/gels, and the exact mechanism underlying gelatin’s helix–coil transition remains under investigation. Despite significant research efforts in this area, only a few studies have directly examined this phenomenon using structural tools like NMR. Here, these postulates were tested using NMR, and our results strongly indicated that the pH-dependent behavior of gelatin is likely solely dependent on the binding of the gelatin either with itself (self-aggregation) or due to binding to the other components of the matrix, rather than the conformational changes of the gelatin molecule itself.

### 3.2 In Situ Adsorption by QCM-D

The interfacial interactions of gelatin with CNC surfaces under different pH conditions were in situ monitored by QCM-D, as shown in Figure 2. In the QCM-D analysis, the changes in frequency are directly related to the changes in mass (i.e., the amount of gelatin adsorbed) on the CNC surfaces, with negative values resulting from an increase in mass. The changes in dissipation, in turn, are related to the viscoelastic properties of the adsorbed material and can also result from high adsorption.

**Figure 2:**
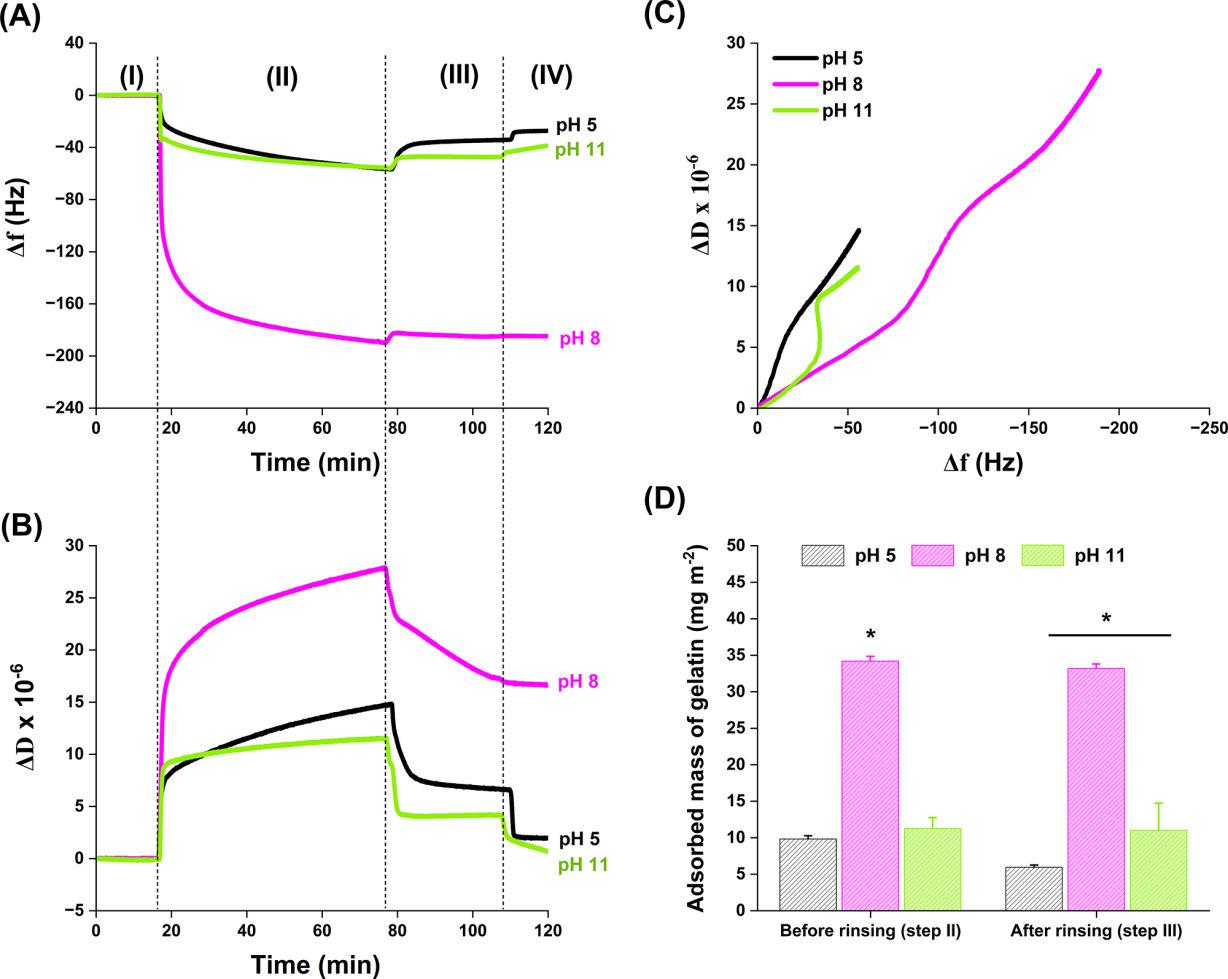
pH dependence of gelatin-CNC interfacial interactions. Changes in (A) frequency (Δ*f*) and (B) dissipation (Δ*D*) as a function of the time during gelatin adsorption onto a CNC surface were measured with the third overtone (15 MHz, *f*_0_ = 5 MHz, *n* = 3) at different pH. In the adsorption process, step (I): baseline obtained by flowing a pH-adjusted water solution, step (II): injection of the 0.5 wt % gelatin solution at different pHs, step (III): rinsing with pH-adjusted water, and step (IV): rinsing with fresh water. (C) D-f plot shows the changes in dissipation as a function of the frequency shift for the third overtone during the first 75 min of QCM-D experiments. (D) Adsorbed gelatin mass calculated according to the Johannsmann model. The third, fifth, and seventh overtones (*n* = 3, 5, 7) were used for the modeling. The asterisk (*) indicates significant differences for *p*-value ≤ 0.05. Plots A, B, and C were generated based on one representative measurement.

In this sense, the changes in frequency (Figure 2A) and dissipation (Figure 2B) during adsorption indicate that a greater amount of gelatin is adsorbed on the CNC surface at pH 8 compared to the other pH conditions. The distinction during gelatin adsorption (step II) is significant: with a ∼190 Hz frequency change, the adsorbed amount at pH 8 is 5–6 times greater than those at pH 5 and pH 11. This could be due to the significant reduction in electrostatic charges on gelatin near its ieP (pH 8), leading to reduced solubility and hydration of this molecule. As a result, less water remains bound to the gelatin, reducing water release during adsorption and allowing more gelatin to effectively bind to the CNC surface. This observation is consistent with previous studies showing that protein adsorption is maximized near the ieP due to reduced water coupling,^35,36^ allowing gelatin molecules to adopt a higher packing density on the CNC surfaces.^37^ Similar behavior has also been reported in studies investigating gelatin adsorption on hydrophobic surfaces.^25^ Meanwhile, the dissipation change is also 2–3 times larger at pH 8 (Δ*D* = 28 × 10^–6^) than the corresponding values for pH 5 and pH 11 conditions directly after the exposure to the gelatin solution (step II). A rearrangement in the adsorbed layer appears to occur at all pH conditions during washing out the excess gelatin (stage III), as the dissipation change decreased (Figure 2B), with the most significant change occurring at pH 8 (50% decrease in Δ*D*). This observation is discussed in Section 3.4 together with the viscoelastic properties of the self-assembled films.

In contrast, the adsorption profiles at pH 5 and 11 showed no notable differences, as the Δ*f* and Δ*D* curves remained virtually unchanged during the adsorption process (step II). This similarity suggests that gelatin adsorbs onto the CNC surface via a similar mechanism under both pH conditions. As an amphoteric polyelectrolyte, gelatin’s surface charge varies with pH (Table 1), which in turn affects its interactions with the negatively charged CNC surface, which also changes their ζ-potential with pH (Table S1, Supporting Information).

Previous studies have suggested that protein adsorption on cellulose surfaces can be driven by hydrogen bonding, hydrophobic effects, van der Waals forces, electrostatic interactions, or a combination thereof.^7,25^ Since gelatin carries a negative and positive charge at pH 11 and 5, respectively, the contribution of electrostatic interactions once gelatin interacts with the negatively charged CNC surface is expected to contribute to the adsorption mechanism. However, QCM-D alone cannot directly confirm the presence or nature of these electrostatic forces. Instead, our findings support the notion that an adsorption mechanism dominated by an entropic driving force promotes gelatin adsorption on the CNC surface, even in the absence of significant changes in Δ*f* and Δ*D* at pH 5 and 11. The release of structured water molecules from the CNC surface and the partial dehydration of gelatin chains during adsorption, together with the release of counterions from both the gelatin and the CNC surfaces, contribute to entropy gain in this system, providing the free energy required to make the adsorption process thermodynamically favorable.^38,39^ These observations are consistent with previous studies on polyelectrolyte adsorption onto charged surfaces,^40–42^ suggesting that in aqueous environments, the entropic contribution from water plays a more dominant role than specific directional interactions. Despite the supporting evidence, the characterization techniques employed in this study do not provide direct indication on the specific role of water in the adsorption process, and further research is needed to clarify the interplay among water molecules and gelatin-CNC interactions as a function of pH.

Another key factor influencing adsorption is the hydration state of the proteins. It is well-known that proteins typically carry a net negative charge at pH values above their ieP, which is expected to result in higher solubility and lower adsorption.^7^ However, in our system, gelatin displayed similar adsorption on the CNC surface at both pH 5 and pH 11. A plausible explanation for this observation could be related to the changes in the ζ-potential of CNCs with pH. Although titration experiments confirmed a high density of carboxyl groups on the CNC surface (see Section 2.2), a significant fraction of these carboxyl groups is expected to be protonated at pH 5, reducing the net negative surface charge of CNCs. This notion is supported by the reduction in ζ-potential of CNCs as the pH shifts from 8 to 5 (Table S1, Supporting Information) and is consistent with previous reports.^20,43^

To further investigate the role of pH on the conformational rearrangements of gelatin at the CNC interface, values of Δ*D* were plotted as a function of Δ*f* (D-f plot) during the first 75 min of the QCM-D experiment (baseline and adsorption steps). This approach eliminates the influence of time on the analysis and allows the study of rearrangements and viscoelastic changes in the layered structure of the adsorbed polymer. As shown in Figure 2C, the adsorption curve at pH 5 showed the steepest slope, indicating that the gelatin layers below the ieP are the most hydrated and loosely packed. The further slight decrease in the slope of this curve suggests that the initially very hydrated layer becomes slightly more compact as adsorption progresses. An opposite behavior to that observed at pH 5 is seen at pH 11, where the slope becomes slightly steeper during adsorption, indicating that the initially packed gelatin layer becomes slightly softer during the process. In contrast, the lowest slope observed at pH 8 confirms the formation of highly packed and least hydrated gelatin layers and further indicates that large dissipations are due to high gelatin adsorption rather than high hydration of the layers. As the D-f plot indicates high packing and low hydration of the layer at pH 8, it is reasonable to assume that adsorbed gelatin molecules mainly account for the large observed frequency shift. Similar to what was observed at pH 11, a softening phase is seen at pH 8, with the slope increasing as the adsorption progressed.

In addition, the masses of the adsorbed gelatin layers on the CNC surface at different pH were calculated using the Johannsmann model, as shown in Figure 2D. Since the measured dissipations are greater than 1 × 10^–6^, the Sauerbrey relation (eq 1) cannot be considered as a valid estimation of the mass of the layers in such cases. A significantly higher gelatin mass (60% mass increase) was adsorbed on the CNC surface at pH 8 compared to the adsorbed gelatin masses at pH 5 and 11, which appear to be similar after adsorption (step II).

Furthermore, the extent of irreversible adsorption, i.e., the adsorbed mass before and after the rinsing step (difference between ends of step II and III), provides information on the effect of pH on the assembly/disassembly of gelatin-CNC thin films. Since the gelatin masses before and after rinsing remain almost similar for each sample, it is suggested that gelatin adsorption on CNC is largely irreversible, with minimal mass loss observed after rinsing. At this stage, only the film assembled at pH 5 showed a slight decrease in gelatin mass after rinsing, suggesting that some proteins are washed off the CNC surfaces during rinsing. The softer nature of the gelatin layer at pH 5, observed in the D-f plot, could support this result. The masses calculated in this study are similar to those reported by Khakalo et al.^35^ for gelatin adsorbed on a cellulose surface at different pH, with values between 9.3 mg m^–2^ and 26 mg m^–2^. A different behavior was observed after rinsing, where the gelatin masses calculated were significantly different (*p*-value ≤ 0.05), suggesting that gelatin interacts with water differently depending on the surrounding pH. This observation is consistent with previous studies stating that water molecules influence the molecular mobility and stiffness of polysaccharide-based films containing moieties such as carboxyl, amino, and hydroxyl groups,^44^ as in the case of gelatin and CNCs.

To further evaluate the potential role of bulk effects on the QCM-D response in this system, we determined the kinematic and dynamic viscosities of gelatin solutions at different pH, as well as the square root of the product of dynamic viscosity and density (Table S2, Supporting Information). Our results show minimal variation in the evaluated parameters, suggesting that viscosity-induced bulk effects on the QCM-D response are negligible in our system. This finding is consistent with a previous study on cellulose substrate reporting kinematic viscosities of a similar order of magnitude.^45^

### 3.3 Ex Situ Analysis of Adsorbed Layers

AFM images obtained before and after gelatin adsorption (Figure 3) reveal no significant morphological differences between the spin-coated CNC film (control material) and the gelatin-adsorbed surfaces. This lack of detectable topographical alteration indicates that gelatin adsorption onto the CNC surface is a case of molecular adsorption, i.e., no presence of discontinuous aggregates or globular structures, as often detected after protein adsorption.^35,46^ The absence of such features supports the hypothesis of a uniform, continuous gelatin layer rather than the formation of distinct domains or phase-separated regions. Particularly, the phase contrast images (Figure 3, bottom row) do not reveal any differences that would imply the inhomogeneity of the gelatin adsorbed layer. The presence of gelatin on the CNCs surface after adsorption is further supported by an increase in the root-mean-square (*R*_q_) values of the films compared to the CNC film before adsorption (*R*_q_ = 1.53). Importantly, the *R*_q_ values of the films after adsorption align with the extent of the adsorption process, i.e. a higher amount of adsorbed gelatin at pH 8 on the CNC surface (Figure 2A) corresponds to a higher *R*_q_ value, suggesting that increased adsorption is accompanied by a measurable increase in nanoscale roughness. This trend is corroborated by earlier studies stating that higher surface roughness may be responsible for higher amounts of adsorbed protein.^1,7,47^ Conversely, the *R*_q_ values observed after adsorption at pH 5 and pH 11 show minimal differences, which also aligns well with the lower and similar adsorption behavior detected by QCM-D under these pH conditions. Despite the direct correlation between QCM-D and AFM results, it is important to note that these measurements were performed on dried films, and the surface roughness may differ in the wet state.

**Figure 3:**
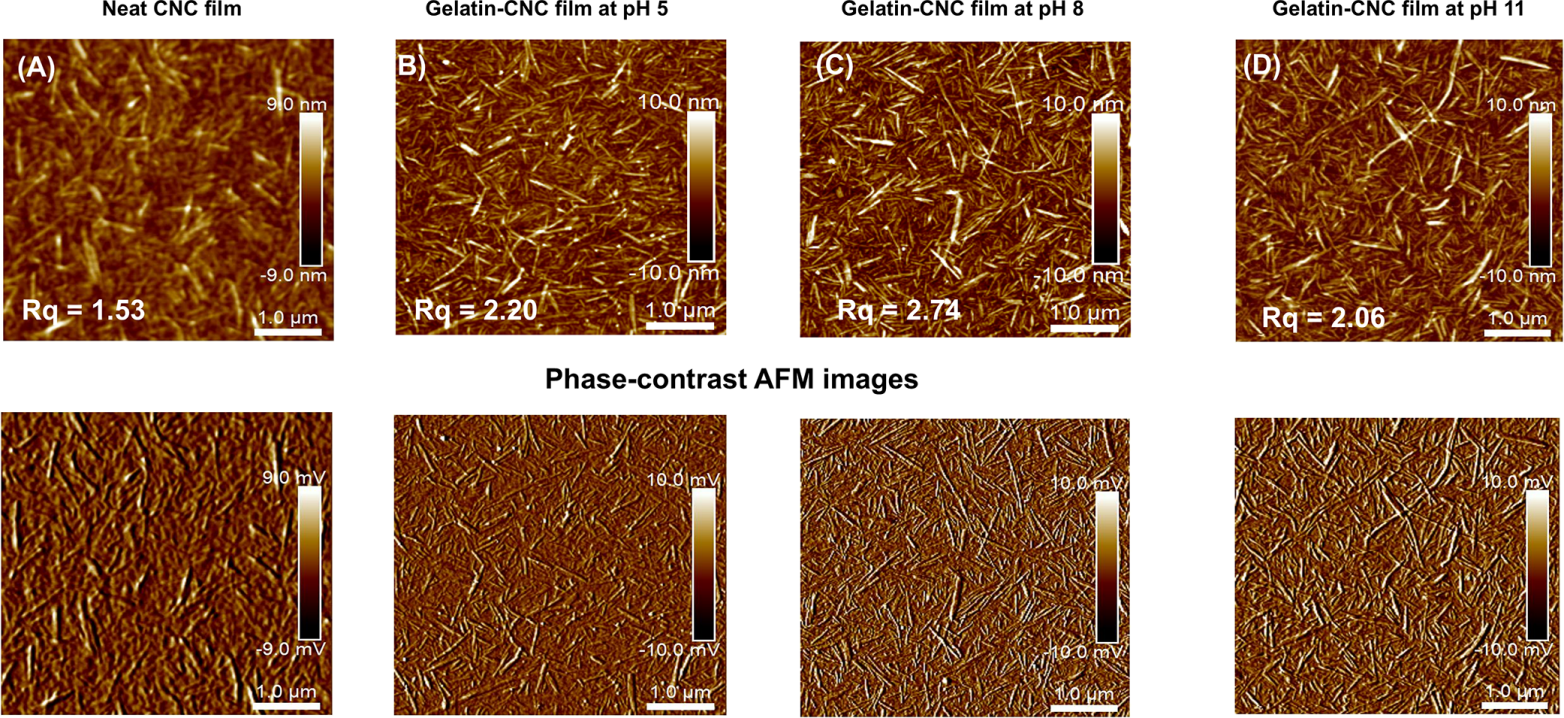
AFM height images (5 × 5 μm^2^) of (A) neat spin-coated CNC film, (B) gelatin-CNC film at pH 5, (C) gelatin-CNC film at pH 8, and (D) gelatin-CNC film at pH 11. Phase-contrast AFM images are included at the bottom of each film. The root-mean-square (*R*_q_) roughness value of three independent AFM images for each film was obtained using NanoScope analysis software (Bruker), and an average value is used in this analysis.

The interfacial chemical composition of the assembled films after adsorption was investigated by XPS and compared to that of the neat CNC film (before adsorption). The carbon (C 1s), nitrogen (N 1s), oxygen (O 1s), and gold (Au 4f) XPS deconvoluted spectra for each sample are shown in Figure S3 and discussed in the Supporting Information. The relative amounts of carbon, nitrogen, oxygen, and gold present in the thin films are summarized in Table 2. The presence of gold (from the QCM sensor) before adsorption in the neat CNC film is likely due to the contribution from the substrate beneath the porous CNC network. After adsorption, all assembled films showed high carbon and similar oxygen content, consistent with the gelatin and CNC chemical composition.

**Table 2:** Relative Concentrations of Carbon, Nitrogen, and Oxygen Detected in Spin-Coated CNC Surfaces and Gelatin-CNC Thin Films

surfaces	carbon (%)	nitrogen (%)	oxygen (%)	gold (%)
neat CNC film	46.2 ± 0.8	-	19.6 ± 0.8	30.7 ± 1.1
gelatin-CNC film at pH 5	46.4 ± 0.7	7.9 ± 0.1	18.2 ± 1.8	27 ± 2.0
gelatin-CNC film at pH 8	56.2 ± 1.4	12.4 ± 1.5	18.6 ± 0.5	12.7 ± 3.3
gelatin-CNC film at pH 11	47.8 ± 0.2	9.6 ± 0.4	18.4 ± 1.0	24 ± 1.1

In this analysis, the nitrogen signal was used as a fingerprint to estimate the amount of gelatin adsorbed onto the CNC surface, while the relative gold content provided information about the surface coverage after adsorption. A reduction in gold signal was seen in all films compared to the neat CNC film, confirming gelatin coverage on the CNC surface. The extent of gold reduction directly correlates with the extent of gelatin adsorption, i.e., the gold content was reduced by 55% when the gelatin-CNC films at pH 8 and 5 were compared. This reduction aligns with the increase in the adsorbed gelatin mass shown in Figure 2D. Similarly, the highest nitrogen content detected in the film assembled at pH 8 aligns with the observation that higher dissipation in QCM-D is due to a significantly higher amount of gelatin adsorbed on the CNC surface (Figure 2D). Conversely, the gelatin-CNC films at pH 5 and 11 showed similar nitrogen contents with slightly higher amount of gelatin at pH 11, which also concurs with the QCM-D data in Figure 2D.

### 3.4 Interfacial Rheology of Gelatin-CNC Thin Films–Voigt Model

The QCM-D data and ex-situ characterization of the surfaces after adsorption showed that gelatin topographical features, coverage, and film roughness were regulated by pH changes to the same extent as the adsorption proceeded. This aligns with previous studies stating that gelatin adsorption on a cellulose surface is strongly pH-dependent ( ^7,25,35^ However, the reason behind the pH-dependency is not conformational changes as shown by NMR data (Figure 1B). Because QCM-D dissipation changes directly reflect energy losses during oscillation, associated with either the viscoelastic properties of materials or higher polymer deposition on a surface, the Voigt model provides a powerful means of linking these dissipation changes to the rheological behavior of the interfacial layers. Therefore, Voigt modeling was undertaken to quantify and illuminate distinctions between the viscoelastic properties of the adsorbed layers.

In our system, the conditions for considering the Voigt model valid are met and supported by Figure 3, with high-quality fittings (Figure S4, Supporting Information). Figure 4 shows the evolution of the shear viscosity (η_*f*_), shear elastic modulus (μ_*f*_), and hydrodynamic thickness (*h*_*f*_) of the different films up to the end of the first rinsing step (step III in Figure 2). The results show an increase in η_*f*_ and μ_*f*_ of the film assembled at pH 8 compared to its counterparts. Similarly, the film assembled at pH 8 showed a greater hydrodynamic thickness than those at other pH conditions. This is likely due to the strongest binding of gelatin on the CNC surface at pH ∼ ieP, consistent with the XPS results. This observation is also supported by the larger hydrodynamic diameter of gelatin at pH 8, as shown by DLS analysis (Figure 1A).

Furthermore, only the film assembled at pH 11 had a more constant hydrodynamic thickness than its counterparts, which could be related to the more stable Δ*f* and Δ*D* signals (Figure 2A,B) compared to the very slow adsorption still occurring at pH 5 and 8. This result is supported by the observations given in the D-f plot analysis, which showed that gelatin layers are less hydrated at pH 11 than at pH 5. Hence, it makes sense that the shear viscosity and shear elastic modulus also reach higher values at pH 11 than at pH 5, as water molecules associated with the adsorbed layers are likely to soften the structure and cause looser packing of the protein molecules on the surface.

During the rinsing process (step III in Figure 2), we observed a decrease in the dissipation changes for all pH conditions, indicative of rearrangement of the adsorbed gelatin molecules on the CNC surface, as mentioned in Section 3.2. Since the shear and elastic moduli increase and the hydrodynamic thickness decreases during rinsing for all pH values (Figure 4), it is suggested that the adsorbed gelatin layers on the CNC surface become more densely packed, i.e. adsorbed gelatin layers densified after adsorption, thereby leading to a reduction in the hydrodynamic thickness of the assembled films. For comparison, a schematic representation of the suggested rearrangement changes of gelatin in solution and once adsorbed onto the CNC interface are shown in Figure 5.

**Figure 4:**
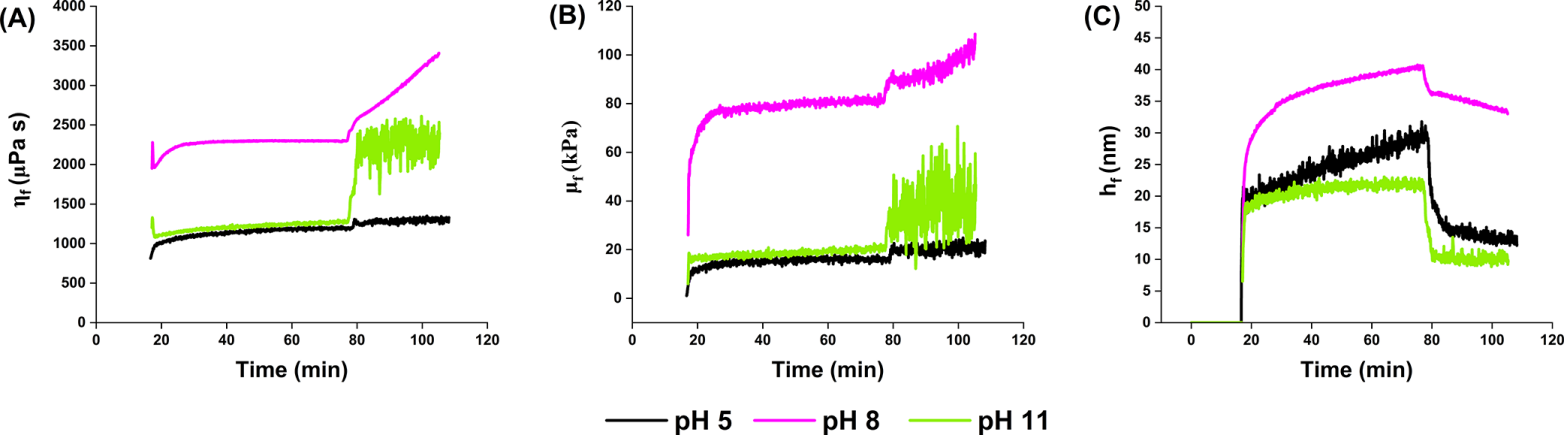
Viscoelastic modeling of gelatin adsorption data. (A) shear viscosities (η_*f*_), (B) shear elastic moduli (μ_*f*_), and (C) hydrodynamic thicknesses (*h*_*f*_) of the different gelatin layers as a function of the time until the end of step III as obtained by fitting the Voigt model to the QCM-D adsorption data. The third, fifth, seventh, and ninth overtones (*n* = 3, 5, 7, 9) were used for the modeling. The plots are based on values calculated for one representative measurement.

**Figure 5:**
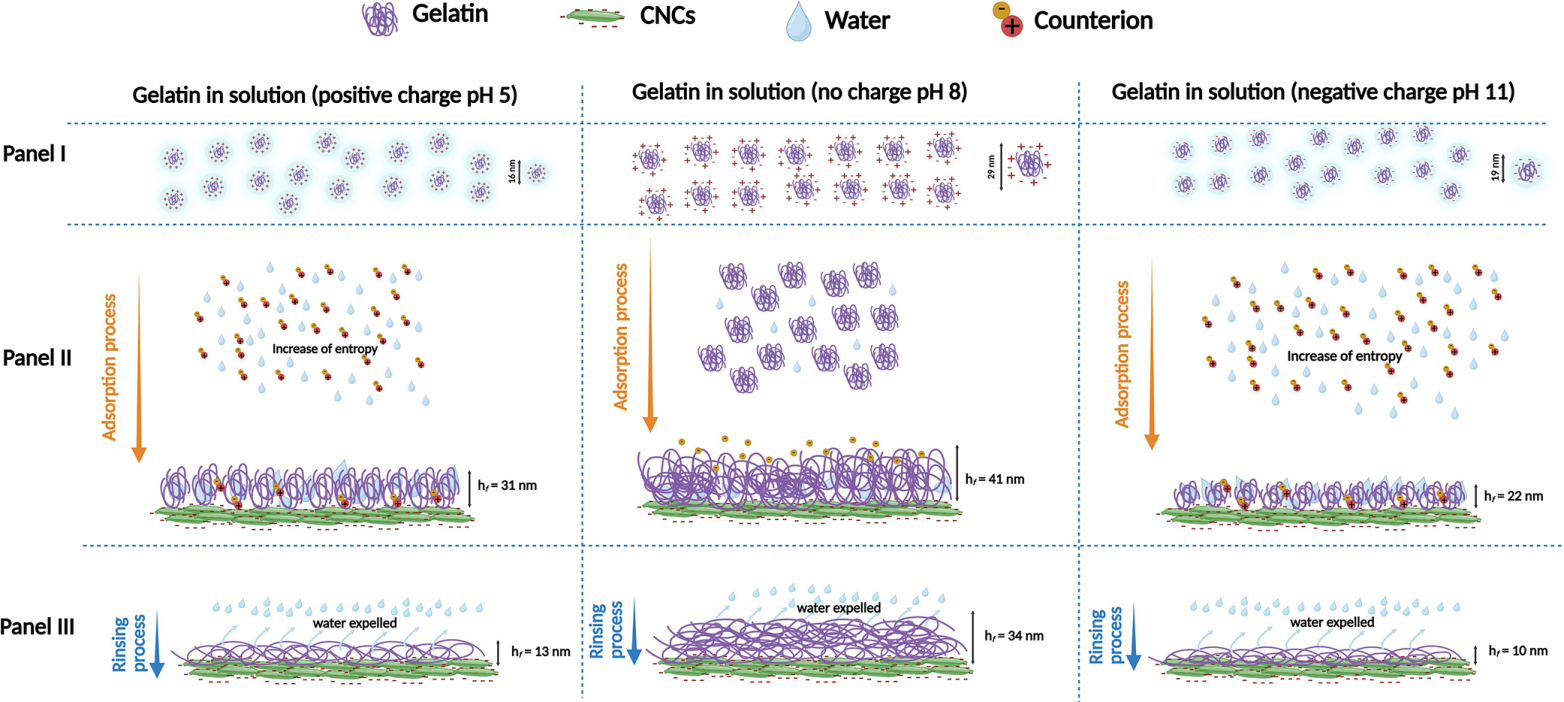
Schematic representation of gelatin molecules in solution (measured by DLS, Figure 1A and Table 1) and at the solid–liquid CNCs interface (measured by the Voigt model) under different pH conditions.

In Figure 5, Panel I illustrates the solution properties of gelatin, with pH-dependent variations in its hydrodynamic diameter. In the adsorption stage (Panel II), the proposed gelatin adsorption mechanism on the CNC surface at different pH was graphically illustrated. Despite that adsorption occurred at all pH conditions, the underlying adsorption mechanisms driving this process have been shown to be pH-dependent. At pH 5 and 11, the adsorption process was driven by entropic forces mainly due to the release of counterions and bound water at the gelatin-CNC interface. In contrast, gelatin adsorption at the ieP (pH 8) primarily occurs due to the closer packaging of gelatin molecules on top of the CNC surface, despite the entropy gain role. These variations in the adsorption mechanism led to variations in the thickness of the adsorbed gelatin layer.

During the rinsing step (Panel III), water effectively removes loosely bound gelatin, resulting in a general reduction of *h*_*f*_ values compared to the values after adsorption (Panel II). The thickest adsorbed gelatin layer was seen at pH 8 before (*h*_*f*_ = 41 nm) and after (*h*_*f*_ = 22 nm) adsorption, indicating that even after rinsing, the formation of denser, more compact gelatin layers remained firmly adhered to the CNC surface.

Altogether, these observations correlate well with the gelatin solution properties evaluated by NMR and DLS, which were further strengthened by AFM showing extended conformation of gelatin chains attached on top of the CNC surface rather than globular structures. Taken together, these results demonstrate that the viscoelastic properties of the adsorbed gelatin layers are governed by pH-dependent conformational changes and surface interactions. The distinct viscoelastic profiles likely arise from the molecular reorientation of the surface-bound gelatin molecules.

## 4 Conclusions

In this study, we investigated the solution properties of gelatin and its interfacial behavior upon adsorption onto CNC surfaces. DLS and NMR analyses revealed that while gelatin’s hydrodynamic diameter and surface charge polarity in solution vary with pH, these changes are attributed to self-aggregation or binding interactions with other polymers rather than conformational changes. Upon adsorption onto CNC surfaces, gelatin exhibited pH-dependent behavior, with electrostatic interactions playing a lesser role compared to typical cellulose-polyelectrolyte systems. After adsorption, the arrangement of gelatin molecules on CNC surfaces showed distinct pH-dependent variations. However, after the rinsing process, all adsorbed gelatin layers densified on the CNC surface regardless of pH, leading to a decrease in the hydrodynamic thickness of the assembled films. Despite the differences observed, our results consistently showed that gelatin—both in solution and when adsorbed onto the CNC interface—maintains an extended chain conformation, with no coil-to-globule transition occurring under any of the pH conditions tested. This understanding allows the fine-tuning of film responsiveness through pH modulation, which is critical for designing advanced materials for applications in biomedicine, sensors, and responsive surface systems.

## Supplementary Material



## Notes

Figure S1. (A) AFM image of carboxylated CNCs, (B) length distribution histogram of CNCs and (C) height distribution histogram of CNCs. The statistical distributions were obtained from a total of six AFM pictures of the same magnification; pH and conductometric titrations protocol; Example of calculation to estimate the true sensed mass of adsorbed gelatin layers using the Johannsmann model; Determination of kinematic viscosities of gelatin solutions; Discussion of the titration results; Figure S2. pH and conductometric titrations of a CNC suspension; Discussion of ζ-potential results for CNC suspensions; Table S1. ζ-potential values of CNC suspensions at different pH; Table S2. Values for the kinematic viscosity and the square root of the product of dynamic viscosity and density of the gelatin solutions; Figure S3. XPS C 1s, O 1s, and N 1s spectra of the gelatin-CNC films after adsorption; Interpretation of the deconvoluted XPS spectra; Figure S4. Voigt model fitting to gelatin-CNC adsorption data; rReferences (PDF)
