# Supplementary material for: Gelatin Adsorption onto Cellulose Nanocrystals Surfaces at Different pH: A QCM‑D Study
Source: Langmuir. 2025 Jun 10;41(24):15319–30. doi: 10.1021/acs.langmuir.5c00795 (PMC12199459; doi:10.1021/acs.langmuir.5c00795)
Supplement: Supplementary file 1 [file la5c00795_si_001.pdf]

Supporting Information

Gelatin adsorption onto Cellulose Nanocrystals Surfaces at Different pH: A QCM-D Study

Jessica Borges-Vilches [1,], Tuuli Virkkala [2], Kristoffer Meinander [1], Ilkka Kilpeläinen [3], Tekla Tammelin [2,*], Eero Kontturi [1,*]*

[1] Department of Bioproducts and Biosystems, Aalto University, FI-00076 Aalto, Finland.

[2] VTT Technical Research Centre of Finland Ltd, VTT, PO Box 1000, FI-02044 Espoo, Finland.

[3] Department of Chemistry, Material Division, University of Helsinki, FI-00560 Helsinki, Finland.

Table of Contents:

Part I: Experimental procedures -- 3

Figure S1. (A) AFM image of carboxylated CNCs, (B) Length distribution histogram of CNCs and (C) Height distribution histogram of CNCs. The statistical distributions were obtained from a total of six AFM pictures of the same magnification --- 3

pH and conductometric titrations protocol --- 3

Example calculation for estimation of true sensed mass of adsorbed gelatin layers -------------- 3

Determination of kinematic viscosities of gelatin solutions -- 5

Part II. Results -- 6

Discussion of CNC titration results --- 6

Figure S2. pH and conductometric titrations of a CNC suspension --------------------------------- 7

Discussion of ζ-potential of CNC suspensions --- 7

Table S1. ζ-potential values of CNC suspensions at different pH --------------------------------- 8

Table S2. Values for the kinematic viscosity and the square root of the product of dynamic viscosity and density of the gelatin solutions -- 8

Figure S3. XPS C 1s, O 1s, and N 1s spectra of the gelatin-CNC films after adsorption ---------- 9

Interpretation of the deconvoluted XPS spectra results --- 10

Figure S4. Fitting of Voigt model to gelatin-CNC adsorption data ------------------------------- 10

References -- 11

Part I: Experimental procedures

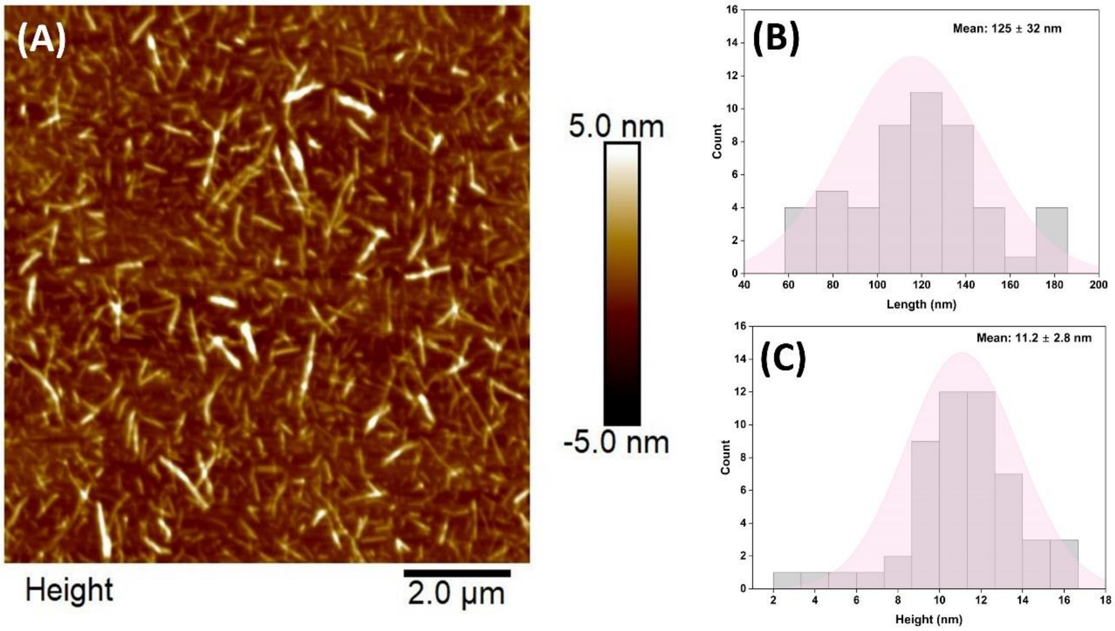

Figure S1. (A) AFM image of carboxylated CNCs, (B) Length distribution histogram of CNCs and (C) Height distribution histogram of CNCs. The statistical distributions were obtained from a total of six AFM pictures of the same magnification.

pH and conductometric titrations protocol: Titrations were conducted to determine the titrant volume necessary to start neutralizing the CNCs. Conductometric titrations were performed as described in section 2.2 of the manuscript and pH titrations were performed using the same CNCs volume (300 mg) in a pH range from 2.6 to 11.3 using 0.01 M NaOH.

Example calculation for estimation of true sensed mass (m^o) of adsorbed gelatin layers: The true sensed mass (m^o) of the adsorbed gelatin layers were calculated according to the model

proposed by Johannsmann *et al.* [1] using the third, fifth, and seventh overtones (n = 3, 5, and 7). The resonance frequencies of the used overtones in Hz are as follows:

Overtone number n:	Resonance frequency f_n (Hz):
3	14870158.61
5	24780958.93
7	34690465.77

The changes in resonance frequency (Δf) during adsorption for each overtone in Hz are obtained experimentally, and multiplying the values by n yields the complex frequency shifts ($\delta^{\wedge}f$):

n:	Δf_n (Hz):	$\delta^{\wedge}f_n$ (Hz):
3	-183.265	-549.795
5	-168.796	-843.98
7	-159.038	-1113.266

Note: The values for one replicate measurement at pH 8 are used as an example of the calculations.

Knowing the following constant values of the quartz:

Density ρ_q = 2648 kg m^{-3}, shear elastic modulus μ_q = 2.9 × 10^{10} kg m^{-1} s^{-2}, and fundamental resonance frequency f_0 = 5000000 Hz, the equivalent masses (m*) for each overtone can be calculated as:

$$m_n^* = -\sqrt{\frac{\rho_q \mu_q}{2f_o} \frac{\delta^{\wedge} f_n}{f_n}}$$

n:	m^*_n:
3	0.000032678

5	0.000030101
7	0.000028363

Values of m* are then plotted against the squares of the resonance frequencies of each overtone:

n:	f_n (Hz):	f_n^2:
3	14870158.61	2.21122×10^{14}
5	24780958.93	6.14096×10^{14}
7	34690465.77	1.20343×10^{15}

By performing a linear fit for the data points, the "y" intercept of the fitted line gives m°, which in this example is 33.2882 mg m^{-2}.

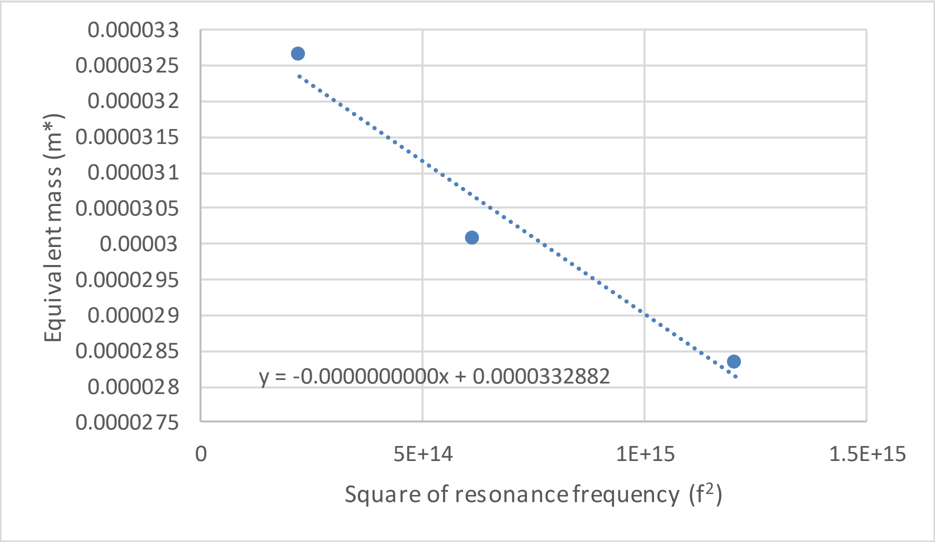

Kinematic viscosity determination: The kinematic viscosity (vs) of gelatin solutions at pH 5, 8, and 11 was measured using an Ubbelohde capillary viscometer at 23°C. The type 53810/I capillary was calibrated using Milli-Q water and Hagenbach and Couette correction factors was used to calculate the real efflux times of the samples. For each sample, the vs values were

calculated based on the corrected efflux times of three replicate measurements using the following equation:

$$\boldsymbol{v_s} = \boldsymbol{K}\,\boldsymbol{t}(\boldsymbol{x})$$

where K denotes the capillary constant and t the corrected efflux time of the sample measured in triplicate.

Using these values, the dynamic viscosity of gelatin solutions at different pH was determined using the following equation:

$$\boldsymbol{\eta_s} = \boldsymbol{\rho_s} * \boldsymbol{v_s}$$

where η_s is the dynamic viscosity of the gelatin solutions and ρ_s = 1.3 g cm^{-3} is the density of the gelatin solution.

Part II: Results

Titration results: The results obtained for each titration as a function of the titrant volume (mmol) are shown in **Figure S2**. To determine the density of negatively charged carboxylate groups on the CNC surface at pH 5, we analyzed the titration curve by linearly fitting the proton concentration data (right y-axis) in the range of 0–0.58 mmol NaOH. From this fit, the volume of NaOH required to initiate the neutralization of CNCs was estimated to be:

$$NaOH\ volume\ (mmol) = \frac{0.523}{0.948} = 0.55\ mmol$$

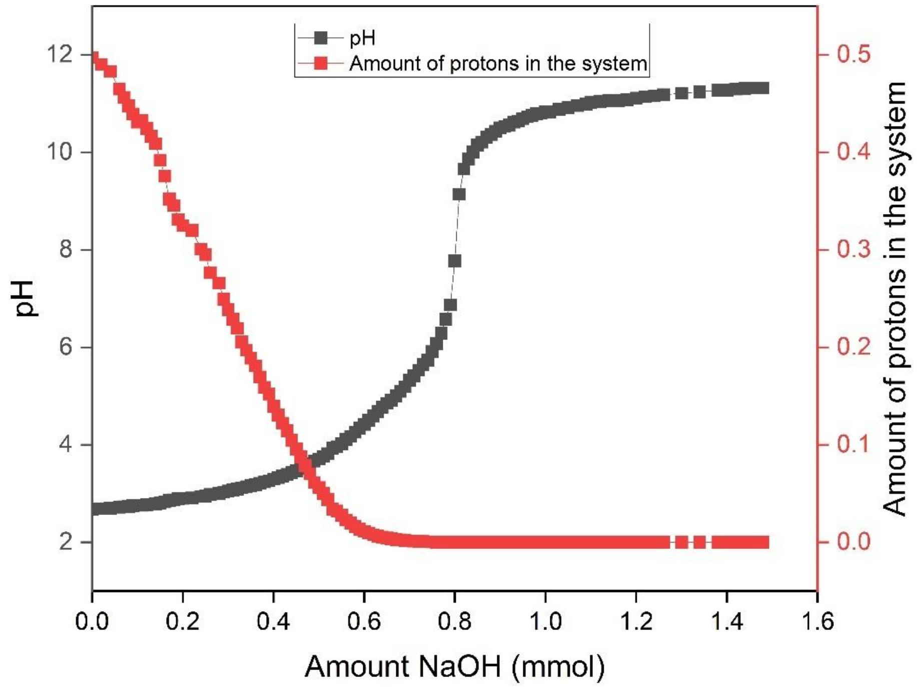

Figure S2. pH and conductometric titrations of a CNC suspension.

This point, corresponding to pH 4.02 (as indicated on the left y-axis), marks the point where the CNCs neutralization starts. The difference between this value and the amount of NaOH required to reach pH 5 (0.67 mmol) represents the amount of deprotonated carboxylate groups present at pH 5. Therefore, the density of negatively charged carboxylate groups on the CNC surface at pH 5 was calculated to be 0.12 mmol NaOH per 300 mg of CNCs.

ζ-potential results: The ζ-potential of CNC suspensions under various pH conditions was measured using a zeta potential analyzer. Table S1 presents the ζ-potential values for CNC suspensions prepared with and without a background electrolyte. As the pH decreased from 8 to 5, a slight reduction in the absolute ζ-potential was observed, indicating partial protonation of the CNC surface at lower pH. As expected, CNC suspensions containing NaCl exhibited lower ζ-potential values than those without added electrolyte, except at pH 11. This deviation may be attributed to the influence of excess protons or hydroxide ions in the solution.

Table S1. ζ-potential values of CNC suspensions at different pH.

	Without NaCl	With NaCl
pH	ζ-potential (mV)	ζ-potential (mV)
5	-45.7 ± 2.0	-42.4 ± 2.3
8	-49.8 ± 1.5	-46.1 ± 1.3
11	-38.2 ± 1.5	-40.2 ± 1.8

Table S2. Values for the kinematic viscosity and the square root of the product of dynamic viscosity and density of the gelatin solutions.

**pH of gelatin solution**	**Kinematic viscosity (10^{-7} m^2/s)**	$\boldsymbol{\eta_s}$ **(10^{-6} mPa s)**	$\sqrt{\boldsymbol{\rho_s \eta_s}}$
5	9.868	1.283	0.001291
8	9.497	1.235	0.001266
11	9.564	1.243	0.001271

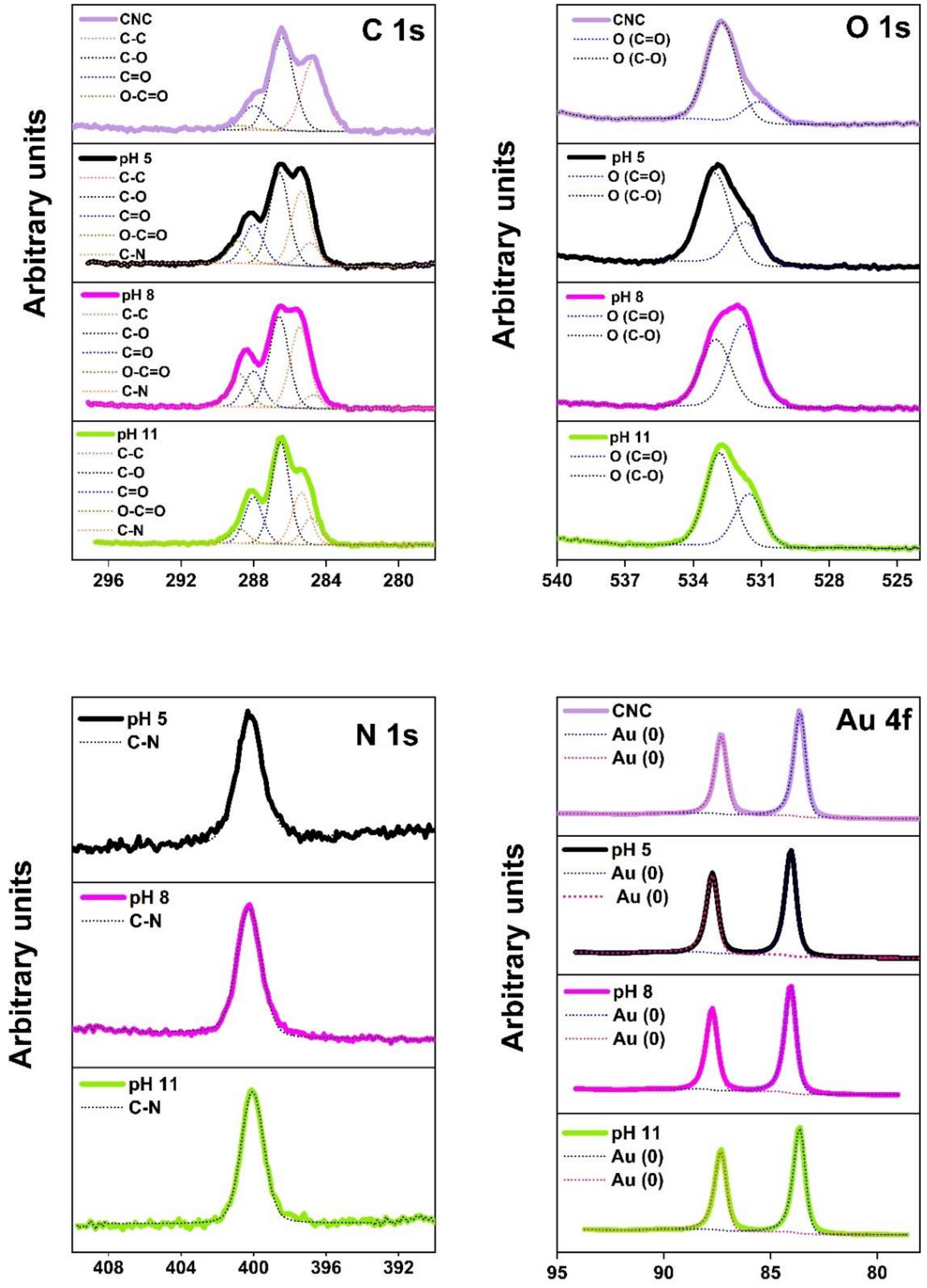

Figure S3. Surface composition of gelatin-CNC thin films after adsorption as a function of pH. XPS spectra were collected to determine the chemical composition of the adsorbed films on the QCM-D sensors. High-resolution XPS spectra from the (A) C 1s, (B) O 1s, and (C) N 1s regions for films adsorbed at different pH.

In all assembled films, the C 1s spectra showed five component peaks: 284.8 eV (C-C), 285.4 eV (C-N), 286.5 eV (C-O), 287.9 eV (C=O), and 288.8 eV (O-C=O). The O 1s spectra showed two components, likely corresponding to C=O (531.4 eV) and C-O (533.0 eV), while the N 1s spectra displayed a single peak at 400.2 eV (C-N bond). Altogether, these results correspond to the typical gelatin and CNC signatures [3,4].

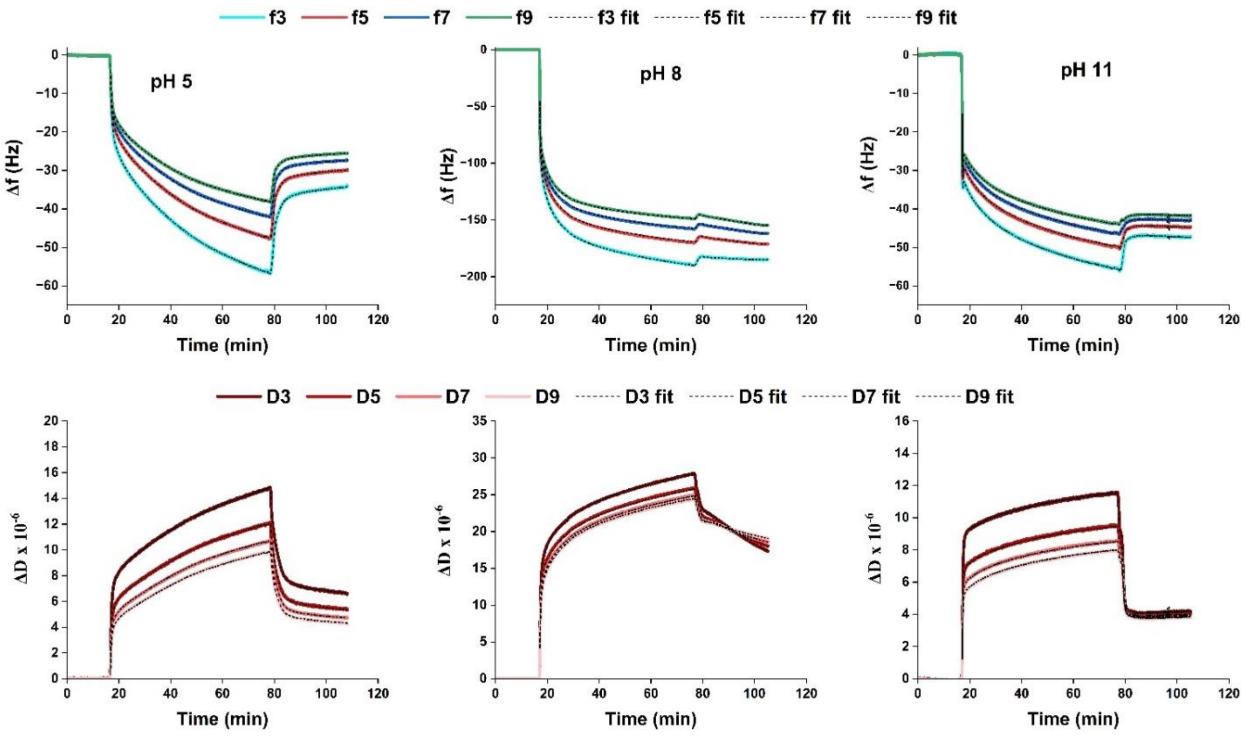

Figure S4. Fitting of Voigt model to gelatin-CNC adsorption data. Δf and ΔD were plotted as functions of time for each pH condition using the third, fifth, seventh, and ninth overtones (n = 3, 5, 7, 9) for the fitting. Solid lines indicate measured QCM-D data and short dash lines the calculated values with the best fit. Fit qualities over 0.8 were obtained in all cases. Layer density was assumed constant as 1.3 g cm^{-3} [2].

References

(1) Johannsmann, D.; Mathauer, K.; Wegner, G.; Knoll, W. Viscoelastic Properties of Thin Films Probed with a Quartz-Crystal Resonator. *Phys. Rev. B* **1992**, *46* (12), 7808–7815. https://doi.org/10.1103/PhysRevB.46.7808.

(2) Khakalo, A.; Filpponen, I.; Rojas, O. J. Protein Adsorption Tailors the Surface Energies and Compatibility between Polylactide and Cellulose Nanofibrils. *Biomacromolecules* **2017**, *18* (4), 1426–1433. https://doi.org/10.1021/acs.biomac.7b00173.

(3) Xu, J.; Li, T.-D.; Tang, X.-L.; Qiao, C.-D.; Jiang, Q.-W. Effect of Aggregation Behavior of Gelatin in Aqueous Solution on the Grafting Density of Gelatin Modified with Glycidol. *Colloids and Surfaces B: Biointerfaces* **2012**, *95*, 201–207. https://doi.org/10.1016/j.colsurfb.2012.02.041.

(4) Boujemaoui, A.; Mongkhontreerat, S.; Malmström, E.; Carlmark, A. Preparation and Characterization of Functionalized Cellulose Nanocrystals. *Carbohydrate Polymers* **2015**, *115*, 457–464. https://doi.org/10.1016/j.carbpol.2014.08.110.
